# Parental experiences of having a child with CLN3 disease (juvenile Batten disease) and how these experiences relate to family resilience

**DOI:** 10.1111/cch.12993

**Published:** 2022-03-04

**Authors:** Mattias Krantz, Emma Malm, Niklas Darin, Kalliopi Sofou, Antri Savvidou, Colin Reilly, Petra Boström

**Affiliations:** ^1^ Department of Psychology University of Gothenburg Gothenburg Sweden; ^2^ Department of Pediatrics Institute of Clinical Sciences University of Gothenburg Gothenburg Sweden; ^3^ Queen Silvia Children's Hospital, Sahlgrenska University Hospital Gothenburg Sweden

**Keywords:** Batten disease, children, CLN3 disease, parents, progressive disease, resilience

## Abstract

**Background:**

CLN3 disease is a neurodegenerative condition presenting in the first decade of life typically leading to death in the third decade. The earliest symptom is rapidly progressive visual impairment followed by intellectual and motor impairments, epilepsy and behavioural disturbances. There are limited data on how the condition affects the family system or the role of family resilience in paediatric neurodegenerative diseases.

**Methods:**

Semi‐structured interviews were conducted with eight parents (five mothers and three fathers) of five children with CLN3. Interview questions focused on the experience of having a child with CLN3, its impact on the family system as well as the concept of family resilience. Data were analysed via thematic analysis.

**Results:**

The thematic analysis resulted in four main themes. The theme ‘recurring losses’ included the feeling of losing a healthy child, the child's loss of abilities and loss of relationships. The theme ‘disruption to the family system’ included that siblings could be ‘side‐lined’, the potential negative impact on romantic relationships and difficulties finding time to oneself. The theme ‘Society is not developed for a progressive disease’ highlighted the difficulties parents faced with respect to contacts with the health and/or social insurance system. The paediatric health care system was seen as supportive, but the adult health care system was not seen as fit for the purpose. Regarding family resilience, parents felt that the disease forced them to reconsider what was important in life. Several parents described that they learned to value small moments of joy and create deep connections through involvement in family routines and rituals.

**Conclusions:**

CLN3 places a very significant burden on the family system including parental feelings of loss, impact on family relationships and lack of understanding within the health/social insurance systems. The concept of family resilience may be useful in understanding the experiences of families affected by paediatric neurodegenerative conditions.

**Key Messages**

- CLN3 disease is a rare neurodegenerative condition presenting in the first decade of life.
- The disease places a significant burden on the family system including parental feelings of loss, impact on family relationships and lack of understanding within the health/social insurance systems.
- Parents felt that the disease forced them to reconsider what was important in life, to value small moments of joy and create deep connections through involvement in family routines and rituals.
- The concept of family resilience may be useful in understanding the experiences of families affected by paediatric neurodegenerative conditions.

## 1 INTRODUCTION

While individual rare diseases (RDs) are by definition of low prevalence (In Europe defined as <1 person in 2000) (https://www.eurordis.org/content/what-rare-disease accessed 5 December 2021), the total number of affected individuals is high, about 3.5%–5.9% of the population (Wakap et al., 2020). Most have neurological manifestations, involving central, peripheral nerve and muscle (Lancet Neurology, 2011). Additionally, most RDs are associated with high unmet needs due to the lack of available and effective diagnosis and treatment measures as well as the relative lack of research to develop such measures (Reinhard et al., 2021). As a response to the challenges of RD, the European Union has established European Reference Networks (ERNs) so that affected individual patients across Europe might benefit from improved diagnosis, care and treatment opportunities. As part of this initiative, patient involvement is emphasized with a focus on active involvement of Patient Advocacy Groups (ePAGs) representatives (Reinhard et al., 2021) highlighting the need to understand the lived experience of patients and their families.

CLN3 disease also known as juvenile Batten disease is a rare neurodegenerative condition caused by a mutation in the Ceroid‐Lipofuscinosis Neuronal 3 gene (Østergaard, 2016). It is part of the neuronal ceroid lipofuscinoses (NCLs), monogenic inherited neurodegenerative disorders that typically present in the first decade of life (Mole et al., 2019). CLN3 disease is believed to the most common form of NCL worldwide. The incidence of CLN3 varies in different countries, with the highest figures having been reported from Scandinavia (Uvebrant & Hagberg, 1997). The incidence was 2.2 per 100,000 live births in Sweden, 4.8 in Finland, 3.7 in Norway, 2.0 in Denmark, and 7.0 in Iceland. Currently, all forms of NCLs are fatal (Mole et al., 2019). In CLN3, treatment is symptomatic and supportive (Bäckman et al., 2005). The children have normal development before disease onset. The first symptoms usually appear at 5–7 years with progressive visual impairment the first symptom in 80% of cases (Østergaard, 2016), followed by cognitive decline, speech impairment, loss of motor skills and epilepsy in early adolescence (Østergaard, 2016). Behavioural difficulties include social, thought and attention problems, aggression and sleep disturbances (Bäckman et al., 2005). Psychotic symptoms, particularly visual hallucinations and delusions, and obsessive–compulsive symptoms have been reported (Adams et al., 2013). Children are wheelchair‐bound by late adolescence and death usually occurs in third decade of life (Østergaard et al., 2011).

In a family system, members strive to maintain balance by using and developing their resources to cope with challenges. A crisis arises when challenges exceed existing resources leading to imbalance. Balance can be restored by the family adapting, acquiring new abilities, reducing the demands they face and creating meaning around their situation (Patterson & Garwick, 1994). The presence of disease significantly affects the family system exposing the system to multiple stressors requiring adaption (Patterson & Garwick, 1994). Progressive diseases such as CLN3 can with time increase the demands on the family system as the child's functionality decreases and the need for care increases (Zurynski et al., 2017). Increasing care needs, a changed perspective on the future, loss of private life and spontaneity and expectations of, or uncertainty around death can lead to increased strains (Stabile & Allin, 2012). The presence of care staff/assistants in the home can further impact on family structure as the nature of their participation in the family may be unclear and changeable (Goldstein & Kenet, 2002; Zurynski et al., 2017). Unpredictable symptoms such as epileptic seizures can further add to uncertainty and stress (Rodenburg et al., 2007).

Family resilience is the capacity of the family to withstand and rebound from stressful life challenges—emerging strengthened and more resourceful (Walsh, 2003). Resilience entails more than managing stressful conditions, shouldering a burden or surviving an ordeal. It involves the potential for personal and relational transformation and positive growth that can be forged out of adversity (Walsh, 2016). Walsh (2003) has identified nine processes organized into three domains that contribute to family resilience (see Supporting Information S1). The processes should be understood as interacting and dynamic where families can mobilize them depending on the challenges they face (Walsh, 2016). The domain ‘belief systems’ includes three processes: making meaning of adversity, positive outlook and transcendence and spirituality. The domain ‘organizational processes’ includes the processes of flexibility, connectedness and mobilize social and economic resources. The domain ‘communication/problem solving’ includes clarity, open emotional sharing and collaborative problems solving.

Research into supporting parental caregivers of children living with life‐threatening or life‐limiting illnesses suggests that caregiver themes include the need to organize basic needs; connect with others; prioritize self‐care; obtain meaningful information; take things day by day; advocate for parental participation; manifest positivity; and celebrate milestones (Smith et al., 2018). This study involved caregivers and professionals of children with a range of life limiting diseases including cystic fibrosis, inoperable congenital heart defects, mucopolyscarridosis, paediatric cancers and severe hypoxic ischemic encephalopathy. Research regarding the specific impact of CLN3 on the family is limited. Schulz et al. (2020) examined the challenges of living with and caring for children with CLN2 disease, another NCL condition. Disease onset is earlier than in CLN3, and main symptoms are language and motor difficulties and seizures (Mole et al., 2019). Families of children with CLN2 report lower quality of life, life satisfaction and lower satisfaction with their partner compared with the normal population (Schulz et al., 2020). We are not aware of any research concerning resilience in the NCLs despite its importance in understanding adaptation to disease. The aim of the current study was thus to investigate parents' experience of having a child with CLN3 and how these experiences can be related to family resilience.

## 2 METHODOLOGY

A qualitative research approach employing semi‐structured interviews and convenience sampling was adopted.

Participants were parents of children diagnosed with CLN3 disease attending or who had attended Queen Silvia's Hospital in Gothenburg, Sweden. Seven children with CLN3 disease were identified, and their parents were contacted by the research team in February/March 2021. Parents of all seven children expressed an interest in taking part. However, the parents of one child were unable to participate during the data collection period (March/April 2021). The parents of another child were not native Swedish speakers, and it was not possible to arrange a translator during the data collection period. Informed consent was provided by all participating parents.

### 2.1 Interviews

Semi‐structured interviews with parents of children diagnosed with CLN3 were developed based on previous literature on parental experiences of having a child with a chronic illness (Boström et al., 2010; Stabile & Allin, 2012). The interview guide (see Supporting Information S2) consisted of open‐ended questions. The questions focused on the parent's experiences of the progress of the child's diagnosis and how it affected the family's everyday life. To capture family resilience, a mind‐map representing the different aspects of Walsh's theory of family resilience (see Supporting Information S3) (Walsh, 2003) was employed during the second part of the interview. The interviews were conducted by authors MK and EM (both master students in psychology) and lasted between 35 and 95 min. Interviews were audio recorded and transcribed verbatim. Due to the Covid‐19 pandemic, interviews were conducted remotely.

### 2.2 Data analysis

Data were analysed according to the six stages of thematic analysis suggested by Braun and Clarke (2006). The approach was abductive, which means that the analysis is not only exploratory but also guided by existing theoretical frameworks.

The transcripts were read repeatedly and subsequently coded both inductively (with a focus on parents' subjective experiences of having a child with CLN3) and deductively (how these experiences related to family resilience as described in the introduction) by authors MK and EM. Data were analysed on a semantic level with a critical realist approach. The coding was discussed with author PB and subsequently revised. Preliminary themes were created by grouping codes, and these were discussed by authors MK, EM, PB and CR and revised until agreement was reached. The themes were confirmed by checking the underlying structure of codes and quotations for each theme.

## 3 RESULTS

Eight parents (five mothers aged 41–51 and three fathers aged 40–51 years) of five children (three male and two female) diagnosed with CLN3 (aged 11–24 years, one deceased; see Table 1 for further demographic information) were recruited. Two fathers did not participate in the interviews.

**TABLE 1:** Descriptive family data

Patient	Participant	Age	Siblings	Child's age at interview	Child's gender	Age at diagnosis	Years since diagnosis
1^a^	Mother 1	48	1	15	Male	7	8
	Father 1	48					
2	Mother 2	41	2	11	Female	9	2
	Father 2	40					
3	Mother 3	52	1	19	Male	8	11
	Father 3	51					
4	Mother 4	42	1	21	Female	9	12
5	Mother 5	56	1	24	Male	6	18

^a^ Deceased.

**FIGURE 1:**
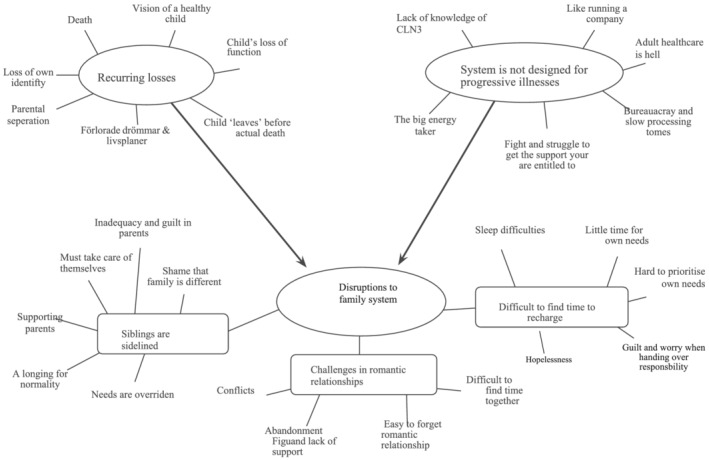
Parenting a child with CLN3 ‐ Themes

**FIGURE 2:**
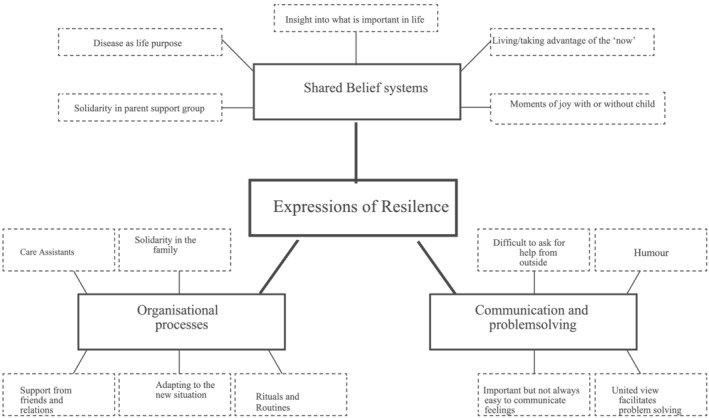
Illustration of the theme ‘expressions of resilience’

The thematic analysis resulted in four main themes: ‘recurring losses’, ‘disruptions to the family system’, ‘society is not created for progressive disease’ (see Figure 1) and ‘expressions of resilience’ (see Figure 2) with six subthemes. The first three themes mainly focused on the difficulties caused by the disease, and the final theme focused on how the family adapted to these strains.

### 3.1 Recurring losses

Parents spoke about recurring losses and grief throughout the course of the disease. The losses began with receiving the diagnosis: “We entered a room with two healthy children and left with one of them dying”. Further losses included the child's loss of function that made everyday living more difficult with less and less time available outside of the family and a loss of relationships with people who used to be close.

Another loss described by parents were the simultaneous losses of losing their own parents while caring for their dying child. Some parents had difficulties making meaning of the losses and described how life was paused because of the child's condition.

All dreams have disappeared. All plans have disappeared. All our dreams in some way, it is not said, but our dreams are in some way linked to the child that is no longer here.

Another loss was that the child's ability to communicate verbally declines markedly. Although alternative ways of communicating were developed, this loss contributed to feelings of losing an important aspect of the relationship with the child. The end stage of the disease with the child completely dependent and with possible psychotic symptoms brings a sense of an additional loss, described by a parent as her child leaving before death had occurred:

During [the child's] final time he had a lot of psychotic symptoms which was horrible, it took over him completely, which led to us losing him more and more maybe half a year before his actual death.

When the child finally died, the parents experienced a loss not only related to their death but also a loss of identity:

That is the hard part now that [the child] no longer exists, that the purpose of my life is gone and that I need to find my way back to something.

### 3.2 Disruptions in the family system

All parents stated that caring for a child with CLN3 was exhaustive and time‐consuming resulting in all other needs being of secondary importance. Parents experienced themselves as inadequate as parents to the sibling/s of the ill child. To maintain balance in the family system, some parents expressed the view that spending time without the affected child was important, not only for the sake of the healthy sibling but also for the couple's relationship. The extensive needs of the affected child could at times lead to frustration and feelings of hopelessness.

#### 3.2.1 Sidelined siblings

All parents with more than one child expressed guilt regarding inadequate parenting and not being present for the healthy sibling. The siblings sometimes acted as emotional support for the parents, as one parent stated: “I have my adult son, he is my ‘rock’, it's awful to say that I have my son for that”. Some parents described how siblings longed for a more ‘normal’ existence and that they often felt shame regarding their sibling's illness including having to live in a home adjusted to accommodate the affected child's special needs.

The siblings could have contradictory feelings and parents pointed to the importance of allowing both positive and negative feelings, although this could be challenging from a parental point of view:

That part is hard, that [the healthy sibling] sometimes hated [the ill sibling] and that it has to be allowed, that he can have these emotions and express these things, and I have to respond to it in an accepting manner.

Some parents expressed positive implications of the illness on the healthy sibling, for instance an increased sense of empathy and increased responsibility:

He has become a much more adult and full human being when compared to peers … It seems that it has made him more responsible, more caring.

#### 3.2.2 Strains in the romantic relationship

Many parents concluded that the extensive caring needs of the child took its toll on the relationship with their partner. All parents described difficulties finding time to spend together as a couple. Parents who lived with the co‐parent described the different roles the parents took on, for instance one parent taking care of the child while the other took care of preparing dinner. Many parents described the fact that one parent always had to be present with the ill child, which for some parents meant they slept in different bedrooms. Some parents emphasized the importance of prioritizing their romantic relationship and the need to make time exclusively for their partners.

One parent described how the small amount of time she had alone with her partner was used to discuss practicalities surrounding the management of the illness, while another parent expressed feelings of abandonment since the diagnosis because of the conflicts and disruptions it brought upon the relationship and family life. Some parents described the difficulties when both parents were feeling stressed at the same time, which led to fewer opportunities to support each other emotionally. For some, there was some solace found in knowing that the other parent was also facing the same stresses. Difficulties in the relationship could arise when the co‐parent was not able to equally participate, cope or take responsibility because of poor mental health.

Sharing and expressing emotions openly were difficult for some of the parents due to lack of time and energy, and for some because of differences between partners regarding the need to express emotions and communicate them to one another. These differences were handled by some parents by talking to friends, family and trusted care assistants or health professionals. One parent described how the differences in emotional openness had led to many conflicts and an emotional shutdown in the family.

#### 3.2.3 Difficult to find time to recharge

A challenge was to make room and find time to recharge and take care of oneself. Many parents felt that there was always something or someone else to prioritize, rather than their own needs. Many parents spoke of the uncertainty that was always present due to not knowing if the supports would work like expected. This could be the case if the child for some reason would not accept support from the care assistant or the assistant would call in sick at the last minute. Some parents described this experience as feelings of having someone else in charge of their life and as a loss of control.

Suddenly an assistant gets sick, and you find no replacement and then we have to jump in, so you are like a prisoner, in your own home, and never know if you can make that trip or dinner or go home to someone or something.

To let someone else take responsibility for caring for the child could be difficult for some parents. Wanting to spend time with their child or wanting to stay close to care for the child in the best way possible if needed was preventing parents from letting others take full responsibility. Meanwhile, it was evident that the time when the child was taken care of by someone else (for instance with extended family) was a source for recovery for the parents.

To tend to one's own needs, like leaving the house to spend time with friends, could lead to feelings of guilt. Another potential obstacle for taking time for oneself was the lack of energy resulting from a lack of sleep and the everyday burden brought upon by the illness.

When you get some time, it's always about that delicate balance deciding whether you have the energy to do something, if it gives more than it takes.

### 3.3 Society is not developed for a progressive disease

The parents experienced the time‐consuming contacts regarding their child's disease as exhausting whether it was health care or the social insurance agency. Health care was regarded as supportive until the child's 18th birthday when the child is moved to adult care. During the child's time in paediatric care, they met a team specialized in the disease. However, in adult health care, they met doctors who knew little about the disease and treated symptoms instead of seeing the whole picture.

Regarding the ‘social insurance agency’, the parents reported long processing times and lack of knowledge about the disease. The system was not adapted for a progressive disease where worsening of symptoms can happen suddenly. Before one application was processed and approved/rejected, they had usually already sent three more applications:

These children should be prioritized in the system and not have to wait in the same way as others. Because that prioritization does not exist, things happen all the time with deteriorations (in function), so that when one thing is done, then it is already too late.

### 3.4 Expressions of resilience

In this theme, parents' experiences are viewed through from a Walsh (2003) model of resilience (Supporting Information S1). ‘Shared belief systems’, ‘organizational patterns’ and ‘communication and problem solving’ were the sub themes in Walsh's model. The focus is mainly on positive adaptations, but difficulties are also addressed.

#### 3.4.1 Shared belief systems

Some parents felt that their child's illness made them see things from a new perspective. They adapted to new circumstances by acceptance and adopting new shared beliefs of what was important in life and thus were able to make meaning of the adversity and adopt a positive outlook. The parents described how they prioritized that the child should experience as much as possible. Meaning was created in many cases through the realization that the family was the most important thing. One parent described the importance of creating participation for the child in the family, albeit in new ways:

Everything takes much longer and it requires more of us but it was like, he [the child] deserved to have it that way, struggling and tiring of course, hard for [the other parent] and me but it was important for us that he should be allowed to participate.

However, other parents felt that it was impossible to create meaning at all in this situation and that life became about perseverance for as long as the child is alive and life could begin again when the child was gone.

Despite the many challenges, several parents described that they learned to value small moments of joy. They mentioned the importance of the child being involved in family routines and rituals despite their limitations. Several parents recounted occasions when they felt a deep connection with their children during the times they shared. These moments could be, among other things, small breaks of calm in the middle of an intense outburst of anxiety, a hug, a smile or a laugh that communicates more than words. One parent described how they saw the disease as their life purpose and that the love for the child made them strong:

I knew he was not with me forever and I was his eyes, I was his voice, I was his, like it was my duty that he should have the best during his time on earth so that was what drove me, the love of [the child] … It really was my life's purpose.

When the child's lost abilities made communication difficult between the rest of the family and the child, remembering shared experiences together became a valuable moment for parents and children:

We have recorded a lot of films and sounds which we still put on inside his room and listen to … it is such a very important thing that you collect memories that you can then pick up, either as sound or, we had experience books for him where we wrote and posted pictures … and so it evokes a bit of memories and joy for him.

Several parents described how important the parental support group for children with CLN3 was to them. In this community, the parents could exist just as they were and be met by others who understand their situation. Sharing knowledge and experiences was a way to create understanding and learn from others.

#### 3.4.2 Organizational processes

In order to be able to live in the present moment and create conditions for the child to participate as much as possible, several parents talked about the need for flexibility and the adjustments they made so that the child can continue to be involved in everyday life:

One of us had to go first and knock with the [ski] poles, then she got a sound that she followed down the slopes. And if that does not work, we have to adapt it …, we have always tried to fight for [the child] to be able to do her things for as long as possible.

With respect to connectedness, some parents brought their child to activities and festivities, while other parents viewed this as an impossibility. All parents made sure to designate time to the healthy sibling, while the sick child was absent. Families described vacations/holidays as an important ritual that was a necessity for recovery and feeling connected as a family.

In terms of social resources, the relief from care assistants and a short‐term stay at a care centre where families of children with disabilities meet were described as a salvation. The care assistants that worked well with the child and family were seen as a valuable support and sometimes parents developed a close bond with the personnel. However, for some parents, the constant presence of personnel in the home made them unable to relax, and it could be difficulty to find continuity in the personnel. Parents viewed support from relatives as a source for relief and resilience although some respondents wanted better understanding from the extended family:

It has been difficult to get other family members to understand how complex this disease is, I should not hide it, but I have not had much help from my other family, I have not, and it can be tough sometimes.

#### 3.4.3 Communication and problem solving

The families differed in how they communicated, some explicitly discussing and some implicit by ‘reading’ each other. Some parents wished for more support from relatives without having to ask for it. Little time was available to explore and talk about feelings. Some families had designated time to communicate feelings to ‘check in’ to see how everyone was feeling. One parent described how the family was a team where the family members balanced between receiving and giving help depending on the situation. A result‐oriented vision including collaborative problems solving within the families and seeing the disease as a common enemy could help with dealing with the grief of the diagnosis:

It is clear that we are different in many ways and not always in agreement, but we have always had the same approach and the same thinking and prioritized the same thing and I can feel that has been our salvation.

## 4 DISCUSSION AND CONCLUSION

This study is the first to our knowledge to examine the experiences of parenting a child with CLN3 and how these experiences relate to family resilience. The theme ‘recurring losses’ highlights the families of children with CLN3 experience recurring feelings of loss during the course of the disease. This feeling of recurring loss or grief has previously been described in accounts of parenting children with chronic illness (George et al., 2007) and meant that parents had to constantly find ways to adapt to new circumstances and sometimes did not have time to adapt until the next loss occurred. At time of diagnosis, parents felt the loss of a healthy child and many of associated hopes and dreams they had for the child. This time of diagnosis and feelings of loss have been identified as often the most difficult periods of a child's illness (Clements et al., 1990). Parents perceive further loss as the child regresses and loses skills across a range of functions. The advanced stage of the child's disease may include the perceived loss of the child's personality as they experience more difficult mental health/psychiatric difficulties. Children with CLN3 can develop symptoms of psychosis and parenting children with psychotic symptoms has been identified as associated with feelings of loss related to the child and the parent (Young et al., 2019). Finally, parents may not only lose to the child to death but also part of their own identity. Previous research suggests that the loss of identity can lead to parents struggling with their sense of competence, mourning the lost parent–child bond and feel a loss of parental hopes for the future (Brotherson, 2000).

The theme ‘disruptions to family system’ includes the increasing care needs of child and how this can challenge the family system. The high care needs reported in this study mirrors that has also been found in parents of children with Sturge–Weber syndrome where it leads to a negative impact on sleep, difficulties to recharge/recover and hard to find time for oneself (Hilbert et al., 2000). In parents of children with CLN3, there is a feeling of failure and guilt regarding the impact of the disease on siblings. Previous research has shown that the greatest negative effect on healthy siblings can be seen in families with children with a chronic illness that requires a lot of time, attention and daily care from parents (Sharpe & Rossiter, 2002). The progressive nature of CLN3 means that the need for care increases, which may mean that siblings are negatively affected by the increased time required for the sick child's care. The impact on romantic relationships between the parents was also broached by parents in the current study and has been noted in previous studies of children with chronic illnesses (Kratz et al., 2009). One of the main reasons for the strain was lack of time together highlighting the need for supports such as respite care to allow both parents to spend time together.

The theme ‘society is not developed for a progressive disease’ highlights that families of children with CLN3 may have to deal with a lack of supportive environment outside of the family. It echoes previous research suggesting that parents of children with chronic illness often find dealing with health and social care systems as the most stressful aspects of their child's condition (George et al., 2007). A family in crisis can function and maintain their well‐being depending not only on how the system mobilizes resources within the family (Ungar, 2016) but also on the supports receives from the wider community. Thus, a perceived lack of support from community resources can impact negatively on the family and parental well‐being. Lack of knowledge among health care professionals of the disease is also an important aspect that the parents in this study highlighted and has also emerged in previous studies of families with children with progressive neurodegenerative diseases (Retzlaff, 2007).

In this study, we found that family resilience theory was applicable to the parents' experiences and that it was possible to identify expressions of resilience. The resilience model consists of nine processes that interact with each other; however, families do not have to mobilize all these processes to be seen as resilient. In terms of ‘shared belief systems’, a crisis can bring together and strengthen the family system through a unified view and insights into what is important and in the current study the parents managed to create meaning in a difficult situation. With respect to organizational processes, the current study suggest that affected families can show flexibility and positively adapt to the new situation. However, mobilizing social and economic resources can be hampered by factors outside the family system such as how well the health and social care mobilize to support the family. Families often perceived these as deficient, and therefore, they became a source of frustration and additional stress instead of promoting family resilience. With respect to communication and problem solving, open emotional sharing and collaborative problems solving were evident but was not possible in all families. It must be noted that it may not be possible to identify expressions of all aspects of family resilience and thus the family resilience approach or some of its aspects may not be a useful lens for all families. Trying to achieve a unified vision and common path in life is difficult and may in some cases be impossible as the challenges become too many and the resources within and outside the family too scarce (Walsh, 2016).

### 4.1 Implications for future research and practice

In terms of supporting families, the results highlight the need for faster processing times at authorities such as the social insurance agency due to the changing disease picture. Results also point to a need for cohesive adult health care, with better communication with families and care staff, as well as increased knowledge about the disease and its impact on the family. It also points to the need for an effective transition to adult care including transfer of knowledge and maintenance of supports. A resilience perspective can allow those supporting families and families themselves to identify sources of support and aid emotional sharing and problem solving.

In order to gain a greater understanding of families' opportunity for resilience, future research may examine in more detail how families are affected at specific phases of the disease process but also in relation to the child's symptoms that can vary significantly (Augestad et al., 2008). As research on the impact of CLN3 on families is still limited, future research should examine larger samples to better understand how the disease affects different family systems and what support measures families may need. Future studies with larger samples are also likely to benefit from the use of a formal measure of family resilience, which would allow quantitative exploration of factors associated with family resilience in families affected by life limiting paediatric diseases. Furthermore, the sibling perspective can be explicitly examined to better understand how all of the family are affected. Finally, future research may also include health and care staff's perspectives to explore how knowledge of the disease and the transition between paediatric care and adult care can be improved.

### 4.2 Study limitations

A significant limitation of the current study is that we interviewed the parents of only five children with CLN3 disease and two fathers did not participate. Additionally, we used a convenience sample, and it is unclear how representative the sample is of families affected by CLN3. With respect to the concept of family resilience, our approach attempted to apply a predetermined theory of family resilience to parental experiences. Our use of a predetermined model can be seen as deductive, as well as more inductive approach for garnering experiences of parenting a child with CLN3 meant that there was some overlap, but this hybrid approach was deemed most suitable for our study aims.

## 5 CONCLUSION

The current study is one of the first to focus on the impact of CLN3 disease on family functioning. Findings suggest that caring for a child with CLN3 disease places a very significant burden on the family system including parental feelings of loss, impact on family relationships and lack of understanding within the health/social insurance systems. Parents experience emotional and practical difficulties during the course of the disease. While taking care of a child with deteriorating function, they must also struggle to ensure that the child receives the supports and care they need from the health and social care system that often seems unprepared for the challenges of meeting the needs of children with progressive disease.

Despite the challenges placed on the family system, it was possible to identify examples of family resilience. Parents can see life from a new perspective and shared memories and beliefs within the family promote resilience. Parents can create new networks with other families who also have a child with a disability. Parents may develop more effective ways of communicating and sharing of emotions.

The results of the current study suggest that the concept of family resilience may be useful in understanding the experiences of families affected by CLN3 and other paediatric neurodegenerative conditions. Future research needs to focus on larger samples and include more formal measures of family resilience.

## CONFLICT OF INTERESTS

The authors have no relevant conflicts of interest.

## FUNDING INFORMATION

This study was supported by the Swedish state under the agreement between the Swedish government and the country councils (ND: ALFGBG‐718681) and Ann‐Mari and Per Ahlqvist Foundation.

## ETHICAL CONSIDERATIONS

The study had been approved by the national ethics committee (no. 2019–05101).

## Supporting information




**Data S1.** Model of Family Resilience (Walsh, 2003)

**Data S2.** Interview protocol

**Data S3.** Mindmap with questions focussing on family resilience

## Data Availability

The data that support the findings of this study are available from the corresponding author upon reasonable request under the heading
