# Supplementary material for: Parental experiences of having a child with CLN3 disease (juvenile Batten disease) and how these experiences relate to family resilience
Source: Child Care Health Dev. 2022 Mar 4;48(5):842–51. doi: 10.1111/cch.12993 (PMC9541062; doi:10.1111/cch.12993)
Supplement: Supplementary file 1 — Data S1. Model of Family Resilience (Walsh, 2003) [file CCH-48-842-s003.docx]

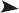


**Belief systems**

**Organizational Processes**

**Communication and problem solving**

Colloborative problem solving

Open emotional sharing

Clarity

Mobilize social and economic resources

Connectedness

Flexibility

Transcendence and Spirituality

Positive outlook

Making meaning of adversity

**Supplement 1**: Model of Family Resilience (Walsh, 2003)
