# Supplementary material for: Parental experiences of having a child with CLN3 disease (juvenile Batten disease) and how these experiences relate to family resilience
Source: Child Care Health Dev. 2022 Mar 4;48(5):842–51. doi: 10.1111/cch.12993 (PMC9541062; doi:10.1111/cch.12993)
Supplement: Supplementary file 2 — Data S2. Interview protocol [file CCH-48-842-s001.docx]

**Supplement 2 -Interview protocol**

Have you any questions before we start the interview?

1. Can you describe who is in your family and talk a little about them?
2. How does a typical day look for your family?
3. If we go back to the time that your child had their first symptom/difficulty, can you talk about this?
4. Can you describe how your contact was with the health system until your child received their diagnosis (of CLN3)?
5. Do you remember how it felt when your child got their diagnosis (of CLN3)?
6. Can you describe how your family was affected by your child’s diagnosis?
7. Can you describe challenges or difficult situations you have had as a family in relation to your child’s condition? (**resilience, follow-up questions - refer to mind map –Supplement 2)**
8. Can you give an example (describe an event) which the family have good memories of?
9. What made this event so memorable for the family?
10. How has it felt to answer these questions?
