# Supplementary material for: Parental experiences of having a child with CLN3 disease (juvenile Batten disease) and how these experiences relate to family resilience
Source: Child Care Health Dev. 2022 Mar 4;48(5):842–51. doi: 10.1111/cch.12993 (PMC9541062; doi:10.1111/cch.12993)
Supplement: Supplementary file 3 — Data S3. Mindmap with questions focussing on family resilience [file CCH-48-842-s002.docx]

How did the disease change you as a family?

How did you get through it?

Did the situation give you new insights?

How did you adapt to the event?

How did you organize yourselves?

Did you cooperate?

## Making Meaning of Adversity

Flexibilit

Have you changed as individuals/as a family?

What did you do to persevere?

Where did you find strength/support?

## Transcendence and Spirituality

Belief Systems

**Resilience**

# Organizational Patterns

## Connectedness

How did you support each other? Practically/emotionally?

How do you deal with individual differences in the situation?

Do you have time and space for individual needs?

How has the disease changed your view of the future?

Did your dreams change?

How did you deal with that?

## Positive Outlook

# Communication and Problem Solving

## Mobilize Social and Economic Resources

Could you make meaning of the disease?

Collaborative problem Solving

Open Emotional Sharing

## Clarity

Did you mobilize relatives/society to deal with the situation?

What was your experience of the support from health care and authorities?

How do you negotiate in hard situations? Did you learn anything from that? How do you make decisions?

Can you share feelings in the family?

**Supplement 3**: Mindmap with questions focussing on family resilience

Has interactions in the family been satisfactory?

How do you show appreciation?

Did you have to change the way you communicated?

How do you deal with uncertain information?
